# Integrative analysis of transcriptomics and proteomics of skeletal muscles of the Chinese indigenous Shaziling pig compared with the Yorkshire breed

**DOI:** 10.1186/s12863-016-0389-y

**Published:** 2016-06-13

**Authors:** Hu Yang, Xing-li Xu, Hai-ming Ma, Jun Jiang

**Affiliations:** College of Animal Science and Technology, Hunan Agricultural University, Changsha, 410128 People’s Republic of China; College of Life Science and Resource Environment, Yichun University, Yichun, 336000 People’s Republic of China

**Keywords:** Shaziling Pig, RNA-seq, Proteomics, Meat quality

## Abstract

**Background:**

The Shaziling pig (*Sus scrofa*) is a well-known indigenous breed in China. One of its main advantages over European breeds is its high meat quality. However, little genetic information is available for the Shaziling pig. To screen for differentially expressed genes and proteins that might be responsible for the meat quality, the *longissimus dorsi* muscles from Shaziling and Yorkshire pig breeds were investigated using an integrative analysis of transcriptomics and proteomics, involving high-throughput sequencing, the two-dimensional gel electrophoresis, and mass spectrometry.

**Results:**

Sequencing produced 79,320 unigenes by *de novo* assembly, and 488 differentially expressed genes in the *longissimus dorsi* muscle of Shaziling pig compared with the Yorkshire breed were identified. Gene Ontology term enrichment of biological functions and Kyoto Encyclopedia of Genes and Genomes analysis showed that the gene products were mainly involved in metabolism, protein binding, and regulation of skeletal muscle development. At the protein level, 23 differentially expressed proteins were identified, which were potentially associated with fatty acid metabolism, the glycolytic pathway, and skeletal muscle growth. Eight differentially expressed genes were confirmed by real-time PCR. These results give an insight into the mechanisms underlying the formation of skeletal muscle in the Shaziling pig.

**Conclusions:**

Certain differentially expressed genes and proteins are involved in fatty acid metabolism, intramuscular fat deposition, and skeletal muscle growth in the Shaziling pig. These results provide candidate genes for improving meat quality and will promote further transcriptomic research in Shaziling pigs.

**Electronic supplementary material:**

The online version of this article (doi:10.1186/s12863-016-0389-y) contains supplementary material, which is available to authorized users.

## Background

Pigs are important domestic animals used for meat production worldwide. Pork quality is influenced by many factors, including breed, nutrition, and post-slaughter handling [1]. Among these factors, the breed is the most important. There are more pig breeds in China than in any other country in the word [2]. In China, 118 indigenous pig breeds are listed as the Domestic Animal Diversity in the World index [3]. Over a long period, breeders have attempted to increase muscle yield and decrease carcass fatness, and great progress for these traits has been made in swine breeding. For example, Landrace pigs and Yorkshire pigs grow more quickly and have higher lean meat than other pig breeds. However, some studies suggest that such intensive selection for increased muscle growth and decreased carcass fatness has led to a deterioration in meat quality [4–6]. Compared with European breeds, Chinese native breeds have higher intramuscular fat (IMF), and increased tenderness and meat quality [7–10]. The Shaziling pig is a well-known breed, which has evolved for centuries in Hunan Province, China, where it feeds on grains, tubers, and wild herbs. It is a Chinese fat-type line with high IMF, superior meat quality, and strong resistance to general diseases. Compared with the Shaziling breed, the Yorkshire has a faster growth rate and a leaner meat ratio.

Skeletal muscle contains several fiber types [11]. Muscle fiber types and the proportion of fiber types affect meat quality directly [12]. Therefore, studies on development and growth are beneficial to improve meat quality. Skeletal muscle development is very complicated, and comprises several stages: determination of myoblasts, proliferation of myoblasts, differentiation and fusion of myoblasts into myotubes and myofibers, and growth and maturation [13, 14]. Analyzing these stages would provide a good basis for understanding muscle fiber development. Previous reports showed that muscle fibers could be classified into red and white fibers [15]. These compositional differences between fibers determine their distinct metabolic type and physiological functions and affect meat quality [16, 17]. Skeletal muscles have been explored extensively using molecular biology [18, 19], and proteomics and transcriptomics techniques have been applied to study the porcine skeletal muscle of different breeds [20, 21].

Over the past decade, proteomic technologies have been used successfully to study skeletal muscle [11, 22]. Proteomic analysis based on two-dimensional gel electrophoresis (2-DE) and mass spectrometry (MS) is a classical method in quantitative proteomics to separate mixtures of proteins into two dimensions and has been a powerful tool in meat science [23, 24]. Many reports concerning differential proteomics among different pig breeds have been published [25–27]. Another technology for characterizing molecular changes in skeletal muscle is analysis of the transcriptome. Recently, transcriptome studies have been applied to analyze differentially expressed genes (DEGs), identify novel genes, describe metabolic pathways, and forecast the relationship between genotypes and phenotypes [28–30]. Next-generation sequencing technology has provided a new tool to quantify transcriptomes and analyze gene expression on a global scale. Transcriptional and proteomic methods could be used to analyze the changes from the mRNA expression to the protein abundance. In addition, post-transcriptional regulation is very important for mRNA stability, translation initiation, and protein stability [31]. Thus, it is necessary to combine proteomic and transcriptional methods simultaneously to analyze skeletal muscle growth and development. Currently, several reports have presented results of both proteomic and transcriptional analyses. For instance, *longissimus dorsi* muscle (LM) proteome and transcriptome profiles of Yorkshire pig and Casertana pig breeds were compared using 2-DE and a microarray. As a result, a large number of genes were identified that are involved in glycolytic metabolism and skeletal muscle growth [32]. In addition, Kim et al. [33] also compared the LM proteome and transcriptome profiles of different pig breeds using 2-DE and a microarray.

In the present study, we performed transcriptomic and proteomic analyses, along with functional enrichment of Gene Ontology (GO) terms and Kyoto Encyclopedia of Genes and Genomes (KEGG) pathway analysis, to characterize the expression profiles in the LM of Shaziling and Yorkshire pigs. The aim of this study was to reveal the differences in breed-related protein and transcript expression patterns between the two breeds. These results could provide an insight into the mechanisms of growth and development of porcine skeletal muscle.

## Methods

### Sample preparation

Three 25-day-old, male full-sibs for each of Shaziling pigs and Yorkshire pigs were slaughtered following the Animal Care Guidelines of the Ethics committee of Hunan Agricultural University. Samples of LM were collected from the same area of the ribs and placed in liquid nitrogen immediately. All samples were kept at −80 °C after collection until used.

### Protein extraction

The frozen LM tissues (100 mg) from Shaziling pigs and Yorkshire pigs were crushed in a mortar containing liquid nitrogen and were then sonicated for 10 s using a Sonoplus (Bandelin Electronic, Berlin Germany). The crushed tissue was homogenized in 1 mL of cold dissolution buffer containing 7 M urea, 2 M thiourea, 1 % dithiothreitol (DTT), 4 % (w/v) CHAPS, 20 μL protease inhibitor cocktail (BBI, Canada), and 2 % (v/v) pharmalyte (pH 3–10; BioRad, Hercules, CA, USA). The homogenate was centrifuged at 15,000 × g, for 20 min at 4 °C. The supernatant fraction was filtered and kept at −80 °C for subsequent analysis. The total protein content was determined using a Bradford assay kit (Bio-Rad).

### 2-DE and images analysis of gel

Approximately 100 μg of extracted protein was diluted with rehydration buffer [8 M urea, 2 M thiourea, 50 mM DTT, 4 % (w/v) CHAPS, and 0.5 % carrier ampholytes (pH 3–10, Bio-Rad)]. The mix was loaded onto 13-cm, nonlinear, immobilized pH gradient strips (IPG, pH 3–10; BioRad), which were rehydrated overnight. After rehydration, first-dimension isoelectric focusing was carried out at 20 °C at 30 V for 12 h, 500 V for 1 h, 1000 V for 1 h, and then gradually increased to 8,000 V. Isoelectric focusing was performed using an Ettan IPGphor III system (GE Healthcare, USA) gel apparatus. The focused IPG strips were incubated for 15 min in equilibration buffer containing 6 M urea, 50 mM Tris–HCl, pH 8.8, 30 % glycerol, 2 % sodium dodecyl sulfate (SDS), and 1 % DTT. Then, strips were then incubated again for 15 min in a buffer containing 6 M urea, 50 mM Tris–HCl, pH 8.8, 2 % SDS, 30 % glycerol, and 4 % iodoacetamide. In the second dimension, the equilibrated IPG strips were placed onto SDS polyacrylamide gels (12.5 % T, 2.6 % C) for protein separation using the Ettan DALT six electrophoresis system (GE Healthcare). When the bromophenol blue dye front reached the bottom of the gel, electrophoresis was stopped and analytical gels were subjected to silver staining and Coomassie blue was used to stain preparative gels before identification by MS. Each sample was analyzed in triplicate.

### Spot choosing and tryptic digestion

Gel images were scanned using an Image Scanner UMax Powerlook 2110XL (GE Amersham) and analyzed using Image Master 2D Platinum software Hofer SE 600 (Version 5.0; GE Amersham). The protein spots were compared automatically using the software, including matching and quality. Spots whose average density was different by more than 1.5-fold between the two pig breeds were analyzed and marked. The differentially abundant spots were cut out from the preparative gel carefully and washed twice with 200 μL of 50 mM ammonium bicarbonate with 50 % acetonitrile and incubated for 15 min at room temperature. The gel pieces were swollen in a digestion solution containing 5 μL of 25 mM ammonium bicarbonate and 10 ng of trypsin at 4 °C for 30 min; in-gel tryptic digestion was run overnight at 37 °C. Subsequently, the supernatant was extracted twice with solvent A (80 % acetonitrile, 0.1 % trifluoroacetic acid) for 15 min at 3 °C. Finally, the digested tryptic peptides were passed through a Zip-Tip to remove salts, according to the manufacturer’s protocol.

### Mass spectrometry

The samples were stored at −70 °C before analysis by MALDI-TOF/TOF. Protein identification was performed using a 5800 MALDI-TOF/TOF mass spectrometer (AB SCIEX) according to the manufacturer’s instructions. Mass spectra were acquired in reflector mode, and recorded in the range of 800–4,500 Da. Eight of the most intense ion signals were selected as precursors for the acquisition of MS/MS. The resulting peptide masses were submitted into the database of the National Center for Biotechnology Information non-redundant (NCBI nr) and the Swiss-Prot database using the Mascot server (Matrix Science, London, UK) to identify proteins. The search parameters were set at ± 100 ppm for peptide-mass mapping (PMF), peptide tolerance and ± 0.4 Da for the MS/MS tolerance.

### RNA isolation and transcriptome analysis

Total RNA was extracted from the LM of the two breeds of the pig using the total RNA extraction kit (Qiagen, Valencia, CA, USA), in accordance with the manufacturer’s instructions. The RNA quantity and integrity were checked using a NanoDrop 2000 spectrophotometer and bioanalyzer 2100 (Agilent Technologies, Santa Clara, CA, USA). A TruSeq RNA Sample Preparation kit v2 (Illumina, San Diego, CA, USA) was used to construct cDNA libraries. Subsequently, the libraries were sequenced using an Illumina HiSeq 2500 instrument (Illumina, San Diego, CA, USA) that generated paired-end reads of 100 bp.

### *De novo* assembling and functional annotation of reads

Raw sequencing reads of each sample were trimmed and assembled *de novo* using CLC Genomics Workbench (CLC Bio, Aarhus, Denmark). After the adapter sequences, ambiguous bases and sequences less than 20 nucleotides were removed, credible contigs generated by *de novo* sequence assembly. The assembled contigs were annotated against the UniProt and NCBI non-redundant (nr) database using BlastX alignment with an E-value cut off of <1e-5. Based on the annotation results, GO terms were extracted using Blast2GO (http://www.blast2go.com) and the results were classified as biological processes, cellular components, and molecular functions. The EuKaryotic Orthologous Groups (KOG) and KEGG databases were used to predict the functions and define the main metabolic pathways, separately.

### Gene expression quantification and differential expression analysis

The expression level of each gene was calculated using the reads per kb per million reads values by the Qualimap v0.5 software [34]. The DEGseq program [35] and R packages were used to identify significantly DEGs between Shaziling pigs and Yorkshire pigs. In this study, the criteria were a fold change (FC) greater than two and cut-off of the false discovery rate of 5 %. For the unigenes that were considered as having differential expression, GO functional enrichment was carried out when the P value was less than 0.05. DEGseq provides statistical routines for determining DEGs.

### Validation of DEGs by quantitative real-time PCR (qPCR)

To validate the expression level of the DEGs, eight DEGs from 2-DE results were analyzed by using qPCR. These genes included Enolase 3 (*ENO3*); ATP synthase subunit beta, mitochondrial (*ATP5B*); Enolase 1 (*ENO1*); Triosephosphate Isomerase (*TPI*); alpha-actin (*ACTA1*); actin, alpha, cardiac muscle 1 (*ACTC1*); myosin light chain, phosphorylatable, fast skeletal muscle (*MYLPF*); and heat shock protein 27 (HSPB1), which mainly belonged to metabolic pathways and structural proteins. Total RNA was extracted from the LM of Shaziling and Yorkshire pigs using the Trizol reagent (Invitrogen, CA, USA), according to the manufacturer’s instructions, and the RNA was treated with RNase-free DNase (Qiagen, Valencia, CA, USA). The quality and concentration of the total RNA was evaluated by gel electrophoresis. The cDNA synthesis of samples was performed using the Quantitect Reverse Transcription kit (Qiagen). The primers (Table 1) were designed by the Roche Universal Probe Library Assay Design Center (https://www.roche-applied-science.com) and synthesized by Sangon Biotechnology Corporation (Shanghai, China). qPCR was performed on an Applied Biosystems 7300 Real time PCR system (Applied Biosystems, USA) with the SYBR Premix Ex Taq Kit (Takara Biotechnology, Japan) in a 20-μL PCR mix. *GAPDH* was used as the reference gene to measure the expression levels of mRNA between the samples and data were calculated by the 2^−ΔΔCt^ comparative CT method [36].

**Table 1:** Primer sequences for the quantitative real-time PCR amplification of the differential expressed genes in Shaziling and Yorkshire pigs

Genes	Primer sequences (5′-3′)	Product size	GenBank sequence no.
HSPB1	F: CGGCAGGATGAGCACGGCTTCA R: GCGCCTCGAAAGTGACAGGGATGG	184 bp	gi|55926209
ENO1	F: GGGGCCTCAACTGGGATCTACGA R: TCCGTGCCGTCCATCTCAATCA	191 bp	gi|753703906
ATP5B	F: CCCTTCTGCGGTGGGTTAT R: CACGGGACAGCACAGTGGTAG	188 bp	gi|89574051
TPI1	F: CAGAGCACCCGCATCATTTACG R: AAGCGCCACCCACAAGGAAC	100 bp	gi|262263205
ACTC1	F: GGGGATGGCGTAACCCACA R: GGCAAGGCATAGCCCTCGTAA	50 bp	gi|545801458
MYLPF	F: GGCGGCAACGTGGACTACAA R: GGCCATCAAAGACCGAAGAGG	94 bp	gi|117660856
ENO3	F: CGGGAAGGACGCCACCAAT R: CCGTTGCGGTAGAACTCAGATGC	165 bp	gi|113205498
ACTA1	F: TCAGGAAGGACCTGTATGCCAACAA R: TGGACAGCGAGGCCAGGATG	186 bp	gi|268607671

## Results and discussion

### Identification of differentially abundant proteins by 2-DE and MS

**Fig. 1:**
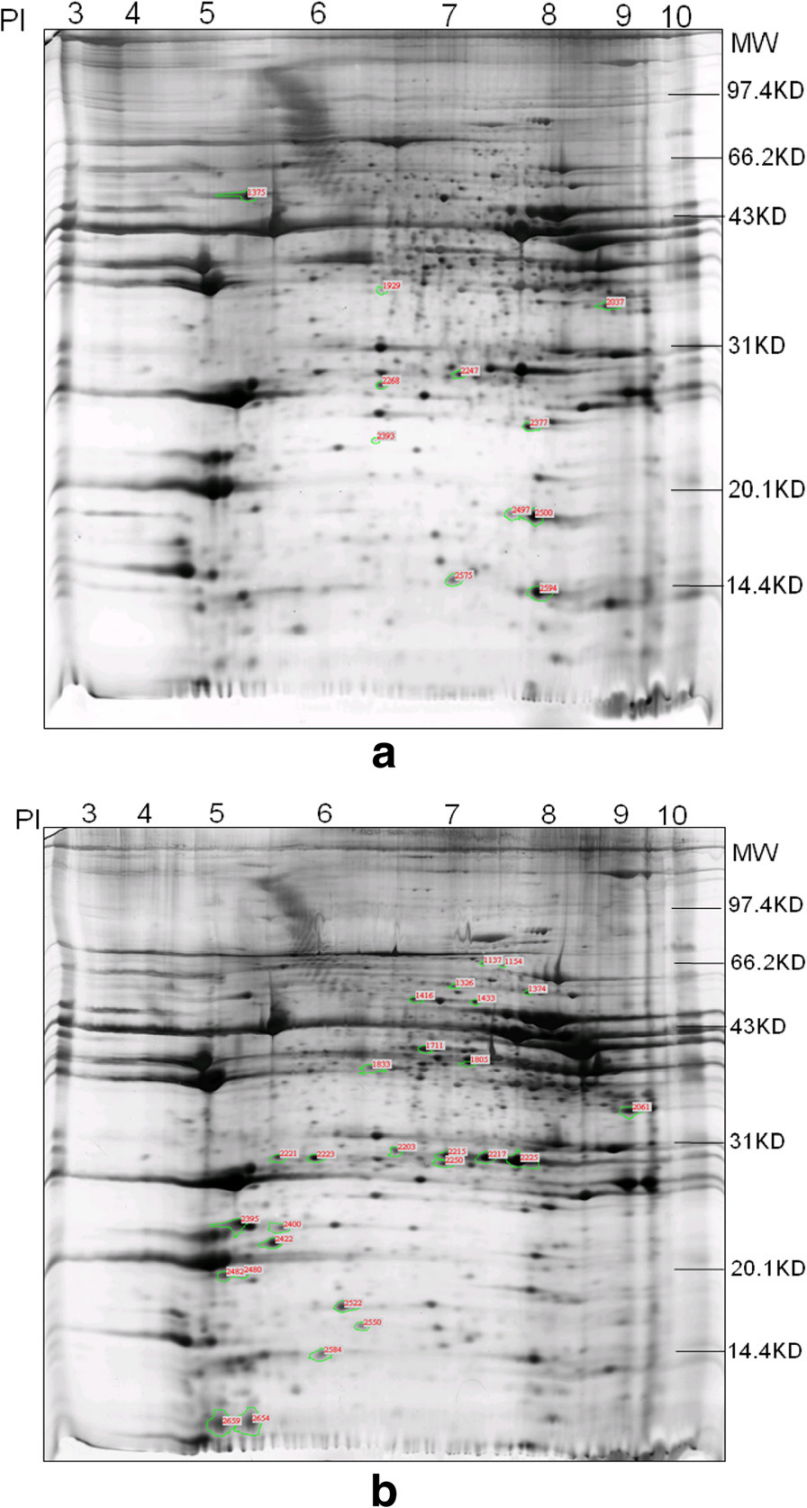
2-DE map of *longissimus dorsi* musle from Yorkshire and Shaziling pig breeds. Scanned 2-DE image of separated using an IPG pH 3–10 strip in the first dimension (12 cm, BioRad, USA), and 12.5 % SDS gel in the second dimension. Tag show 38 spots that were significantly changed between the two breeds [**a** Yorkshire pigs (up-regulation), **b** Shaziling pigs (up-regulation)]

The *Longissimus dorsi* proteomes from the two pig breeds were analyzed by the 2-DE technique. Thirty-eight protein spots (Fig. 1) were significantly different (FC >1.5) between the two breeds: 27 protein spots were upregulated in Shaziling pigs and 11 in Yorkshire. The differentially abundant protein spots were identified by MALDI-TOF/TOF-MS: two spots could not be identified successfully; however, the 36 remaining spots were identified by matching peptide data to the UniProt database. The characteristics of the identified proteins and the identification parameters are listed in Table 2. Some of the identified proteins were resolved as multiple spots. For example, spots 2480, 2654, and 2659 represented the same protein and showed similar quantitative trends. Ultimately, 23 different proteins were identified.

**Table 2:** Protein differentially expressed for Shaziling pig breeds and Yorkshire were identified by 2-DE and MALDI-TOF-MS/MS

Sport no	NCBI accession number	Protein name	Mr, kDa theor	pI theor	No. of peptides identified	Mascot score	Sequence coverage	Expect^e^
Up-regulated in Yorkshire pigs								
1375	gi|89574051	mitochondrial ATP synthase, H^+^ transporting F1 complex beta subunit, partial, ATP5B	47059.6	4.99	16	1180	46 %	5.8e-11
1929	gi|283993079	L-gulonate3-dehydrogenase	35433.2	5.79	14	417	51 %	1.2e-03
2268	gi|545835136	PREDICTED: NADH dehydrogenase ubiquinone flavoprotein 2 isoform X1	25812.2	6.96	14	435	60 %	1.8e-039
2247	gi|55926209	heat shock protein beta-1	22984.7	6.23	9	423	41 %	2.9e-03
2393	gi|809283	Chain B, Structure Determination Of Aquomet Porcine Hemoglobin At 2.8 Angstrom Resolution	16082.4	6.76	12	511	86 %	4.6e-04
2377	gi|545848507	PREDICTED: alpha-crystallin B chain isoform X2	20116.4	6.76	9	212	51 %	3.7e-01
2497	gi|494389	Chain A, High Resolution X-Ray Structures Of Pig Metmyoglobin	16901.8	6.5	9	529	59 %	7.3e-04
2500	gi|494389	Chain A, High Resolution X-Ray Structures Of Pig Metmyoglobin	16901.8	6.5	12	596	82 %	1.5e-05
2575	gi|55926217	cytochrome c oxidase subunit 5B, mitochondrial precursor	14002	8.8	8	334	46 %	2.3e-02
2594	gi|809283	Chain B, Structure Determination Of Aquomet Porcine Hemoglobin At 2.8 Angstrom Resolution	16082.4	6.76	9	400	67 %	5.8e-03
Up-regulated in Shaziling pigs								
2480	gi|117660856	MYLPF	19.0	4.8	6	110	36 %	5.8e-00
2395	gi|117660874	MLC1f	21018.6	4.9	11	444	60 %	2.3e-40
2654	gi|117660856	MYLPF	19066.3	4.8	9	347	49 %	1.2e-03
2422	gi|117660890	MLC3f	16761.2	4.6	4	250	29 %	2.3e-02
2659	gi|117660856	MYLPF	19066.3	4.8	6	144	27 %	2.3e-01
2400	gi|545858131	keratin, type I cytoskeletal 10 isoform X2	58318.6	4.9	16	190	21 %	5.8e-01
2482	gi|117660856	MYLPF	19066.3	4.8	16	600	86 %	5.8e-05
2522	gi|117660874	MLC1f	21018.6	4.9	8	190	40 %	5.8e-01
1154	gi|545845559	PREDICTED: fibrinogen beta chain isoform X2	50399.8	7.94	18	gi|545845559	43 %	4.6e-03
2225	gi|262263205	triosephosphate isomerase 1	26878.9	6.54	14	463	74 %	2.9e-04
2203	gi|262263205	Triosephosphate somerase 1	26878.9	6.54	17	774	77 %	2.3e-07
1326	gi|311247991	PREDICTED: pyruvate dehydrogenase protein Xcomponent-like isoform	54036.4	8.3	12	317	28 %	1.2e-02
1416	gi|545801458	PREDICTED: actin, alpha cardiac muscle 1 isoform X1	42334	5.2	10	313	31 %	2.9e-02
2250	gi|262263205	Triosephosphate isomerase 1,TPI1	26878.9	6.5	17	795	82 %	1.8e-07
2584	gi|314907119	A-FABP adipocyte fatty acid-binding protein	14780.6	6.2	8	371	56 %	4.6e-03
1433	gi|545833443	PREDICTED: alpha-enolase isoform X1	38172.7	8.93	12	377	41 %	1.2e-03
2221	gi|55926209	HSPB1 heat shock protein beta-1	22984.7	6.23	8	236	31 %	1.5e-01
1805	gi|545859898	PREDICTED: beta-enolase isoform X1	48154.9	8.52	17	414	40 %	2.3e-03
2550	gi|545805333	PREDICTED: 14 kDa phosphohistidine phosphatase isoform X2	14036.8	5.91	14	454	74 %	2.3e-04
2217	gi|262263205	triosephosphate isomerase 1	26878.9	6.54	16	701	82 %	4.6e-06
1711	gi|46389777	troponin T fast skeletal muscle type	30720.1	8.68	14	423	41 %	2.9e-03
1137	gi|545845559	PREDICTED: fibrinogen beta chain isoform X2	50399.8	7.94	15	289	34 %	7.3e-02
2215	gi|262263205	triosephosphate isomerase 1	26878.9	6.54	18	684	76 %	2.3e-06
2223	gi|55926209	HSPB1 heat shock protein beta-1	22984.7	6.23	10	371	42 %	4.6e-03
1833	gi|545832797	PREDICTED: troponin T, slow skeletal muscle isoformX1	32449.6	5.54	9	418	32 %	9.2e-03
1374	gi|268607671	actin, alpha skeletal muscle	42366	5.23	11	348	35 %	9.2e-03

^e^Number of times we would expect to obtain an equal or higher score by chance

### Function analysis of differentially expressed proteins

**Fig. 2:**
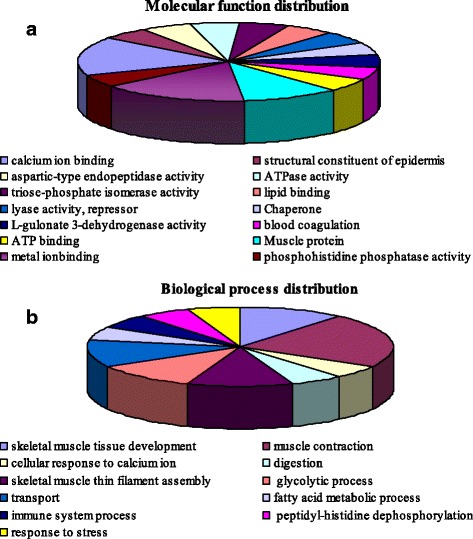
GO annotation of different proteinic spots (**a**: molecular function distribution, **b**: Biological process distribution)

To further determine the functions of the differentially abundant proteins, functional categorization of all the identified proteins was performed using GO annotation. The annotated results of biological process are shown in Fig. 2. The 23 differential proteins were grouped into 11 categories. We focused on the categories of fatty acid metabolism, glycolytic pathway, and skeletal muscle growth and development. The result of enrichment analysis revealed that some of the identified proteins had an important impact on meat quality. For example, TPI plays an important role in ensuring immediate equilibration of the triosephosphates produced by aldolase in glycolysis, which is interconnected with lipid metabolism and to the pentose phosphate pathway [37]. In addition, a previous report showed that a quantitative increase in TPI was positively correlated with more tender meat [38].

Enolase 3 was another metabolic enzyme that was differentially abundant between the two breeds. Enolase 3 is a crucial enzyme in glycolysis that catalyzes the interconversion of diphosphoglycerate and phosphoenolpyruvate. Pig muscle Enolase 3 was investigated some time ago [39]. Many isoforms of Enolase 3 have been confirmed to have an influence on IMF in pigs [20, 40].

Muscles are composed primarily of different muscle fibers. Muscle fiber type is an important factor influencing meat quality [41]. For example, increasing the percentage of type IIb fibers could lead to the reduced meat quality because of altered metabolic rates and biochemical processes [42]. ACTA1 is a member of the actin family and is a major constituent of the contractile apparatus in skeletal muscle. Previous reports showed that alpha-actin levels positively correlate with the synthesis of muscle fiber proteins and muscle growth [43]. In this study, ACTA1 was differentially abundant between the two pig breeds.

Heat shock proteins (HSPs) including HSPA1, HSPA8, HSPB1, and other chaperone proteins have been associated with pig meat quality [44, 45]. In the present study, HSPB1 was more abundant in the Shaziling pig compared with the Yorkshire pig. This observation suggested that HSPB1 is correlated with meat quality. In addition to tenderness, further research is needed to confirm whether the IMF content of meat is affected by the expression of HSPB1.

### Illumina sequencing and *de novo* assembly

**Fig. 3:**
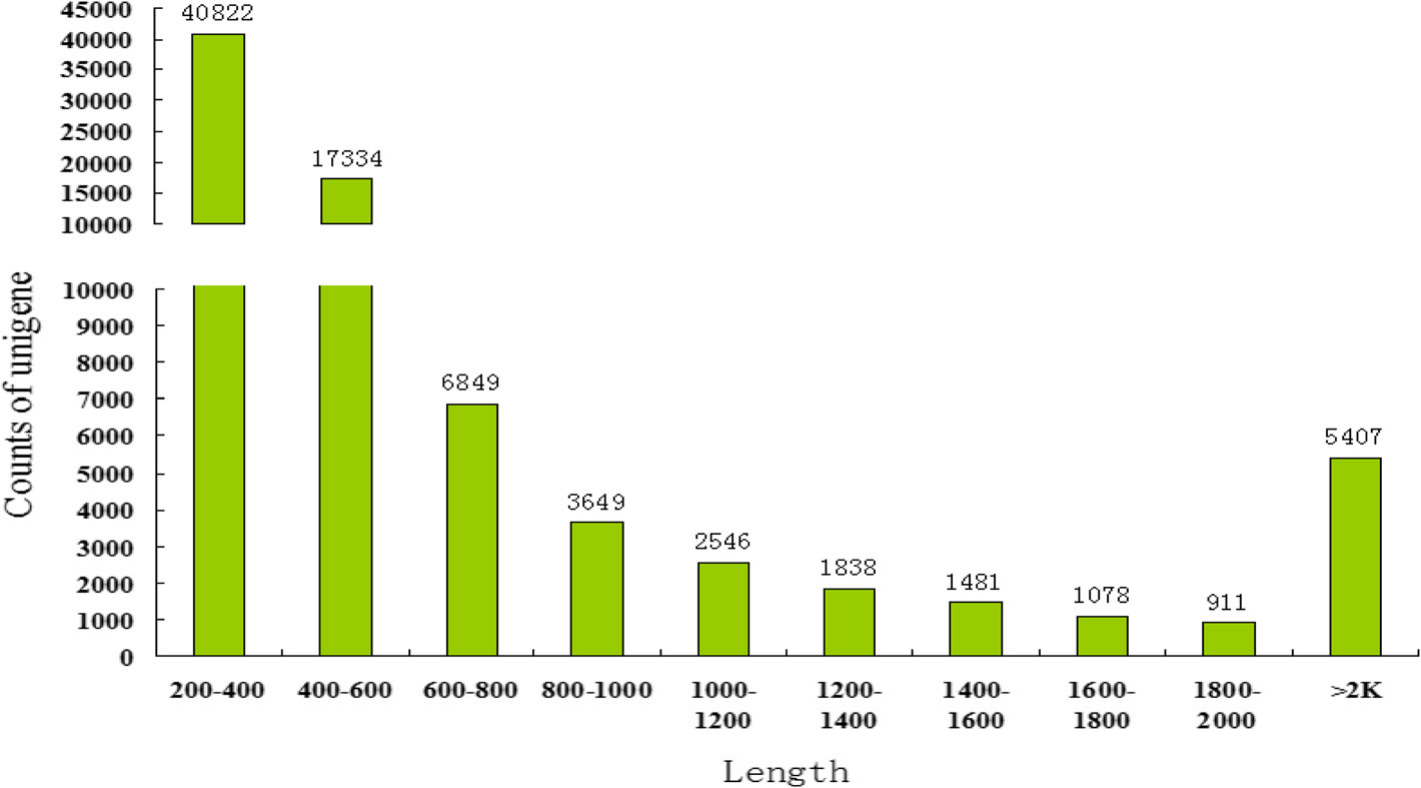
Length distribution of the assembled final unigenes of Illumina sequences

Sequencing generated 30,751,992 and 35,933,274 100-bp paired-end reads from cDNA libraries of Shaziling and Yorkshire pigs, respectively. After trimming of low-quality reads and the removal of adapters, 29,522,984 and 34,937,710 reads were used for *de novo* assembly. The reads were assembled into 86,759 contigs (N50 = 939 bp) ranging from 100 to 49,881 bp, with an average length of 672 bp. Thereafter, the contigs were assembled into scaffolds (N50 = 1028 bp) with a mean length of 713 bp. After the final assembly of scaffolds using CAP3 [46], we obtained 79,320 unigenes (N50 = 1112 bp) with an average length of 733 bp and a maximum of 66,767 bp. The length distribution of the assembled unigenes is shown in Fig. 3. The result of the assembly is shown in Table 3. The transcriptome data have been submitted to the NCBI GEO database under accession number GSE70673.

**Table 3:** Statistical summary of the *longissimus dorsi* muscle transcriptome for assembling

Statistics	Counts	Average length (bp)	N50 (bp)	Longest length (bp)	Total length (bp)
Contigs	86,759	672	939	49,881	58,319,316
Scaffold	81,915	713	1028	66,767	58,377,616
Unigenes	79,320	733	1112	66,767	58,152,234

### Functional annotation of *longissimus dorsi* transcripts

To validate and annotate the protein functions of the 79,320 assembled unigenes, they were searched against the NCBI nr and UniProt protein database using the BLAST program (E-value cutoff <1e^−5^). Of the 79,320 assembled unigenes, 23,055 sequences (29.07 %) were assigned to the nr database, and 25,784 sequences (32.50 %) were found to have significant similarity with proteins in the UniProt database (Table 4). Approximately 30 % of all assembled unigenes was perfectly matched to the Uniprot database and the nr database, respectively. Thus, most of the assembled unigenes were unknown, indicating that many new genes and non-coding RNA sequences were obtained. Generally, direct sequencing of transcriptomes is considered an effective tool to discover new genes [47, 48] and has been applied to various organisms [49, 50]. These unigenes provided much valuable information for further identification of genes related to development of the *longissimus dorsi*.

**Table 4:** Functional annotations using transcript BLAST analyses

Public database	Hit unigenes number	Percentage (hit/total) %
Annotated in nr	23,055	29.07
Annotated in UniProt	25,784	32.50
Annotated in GO	23,702	29.8
Annotated in KOG	16,171	20.3
Annotated in KEGG	16,755	21.1

### Functional classification of unigenes

**Fig. 4:**
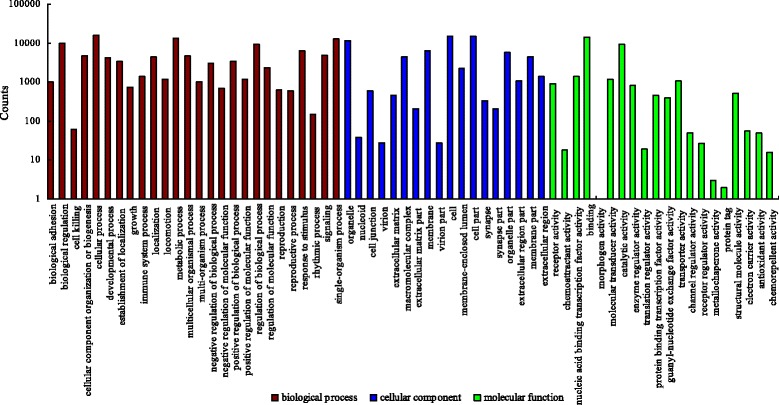
Column chart presentation of GO classification of unigenes

GO is an extremely useful tool to classify the functions of a large quantity of genes, and is an international standardized gene functional classification system. GO analysis has been widely applied to predict the functions of genes in many organisms. Three ontologies, cellular component, molecular function, and biological process, are shared in the GO database. In this study, of the annotated unigenes, 23,702 could be assigned to the biological process (BP, 111,204 sequences), cellular components (CC, 68,072), and molecular functions (MF, 30,087) (Fig. 4) using the Blast2GO program [51]. In the biological process, the majority of unigenes were involved in catalytic activity and transcription factor activity, indicating that they might play a key role in the regulation of skeletal muscle development. In cellular components, organelle, membrane, cell, and cell part were prominently represented. For molecular functions, “catalytic activity” and “binding” represented the majority of the category.

**Fig. 5:**
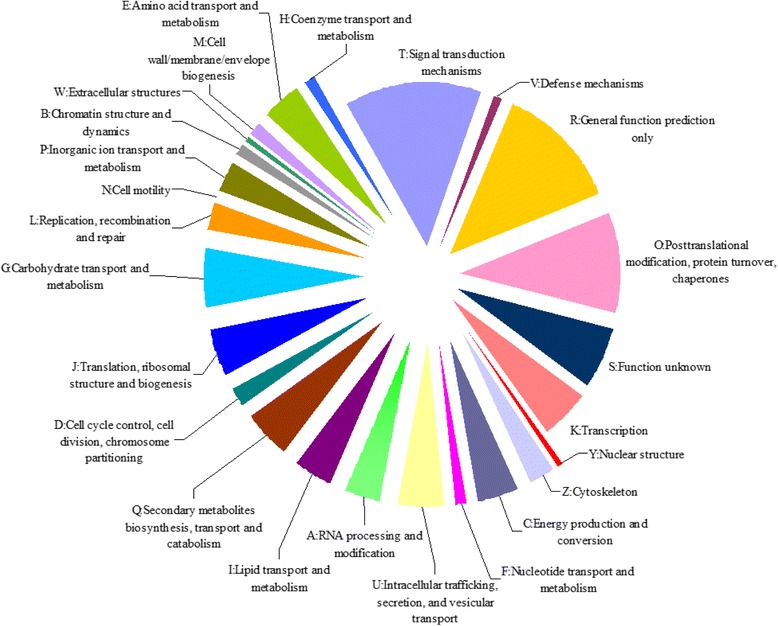
KOG functional classification of transcriptome

To further evaluate the function of the unigenes, KOG classification of all the unigenes was performed, and 16,171 unigenes could be assigned to 25 categories (Fig. 5). Among the 25 KOG categories, the highest proportion of unigenes matched to “signal transduction mechanisms” (13.8 %), followed by cluster for “general function prediction only” (12.6 %), “post-translational modification, protein turnover, chaperones” (9.8 %), and “carbohydrate transport and metabolism” (5.8 %). The KEGG pathway database is a powerful tool for the analysis of gene function in terms of gene networks [52]. To identify the biological pathways of transcriptome from the LM, all unigenes were submitted to KEGG pathway analysis. A total of 16,755 unigenes were assigned to 278 KEGG pathways (Additional file 1: Table S1), which fell into five main categories, including metabolism, environmental information processing, genetic information processing, cellular processes, and organism systems. Among the five main categories, the metabolism pathway represented the largest number of the unigenes (1013). These metabolic pathways were mainly carbohydrate metabolism, energy metabolism, amino acid metabolism, and lipid metabolism, which provided a valuable resource to investigate muscle growth and lipid metabolism processes.

### Identification and analysis of DEGs

**Fig. 6:**
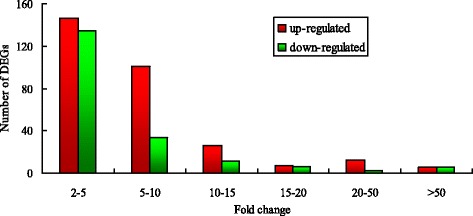
The fold change distribution of up- and down-regulated DEGs. Green bars refer to down-regulated DEGs and red bars refer to up-regulated DEGs in Shaziling pigs compared with Yorkshire pigs. The X axis shows fold change of DEGs and the Y axis number of DEGs

To identify the DEGs between the Shaziling and Yorkshire pigs, the relative expression of the genes was estimated using the RPKM value, which is a common method for estimating transcript levels [53]. According to the RNA-seq analysis, DEGs were selected using the criteria of FC ≥2 and false discovery rate ≤0.05; the FC distribution of DEGs is shown in Fig. 6. A total of 488 unigenes were identified as DEGs between the two breeds, of which 297 were upregulated in the Shaziling pig and the 192 genes were upregulated in the Yorkshire pig. To better explore the functions of the DEGs, GO functional analysis was carried out. The results indicated that 488 DEGs were significantly enriched in 208 GO categories (Additional file 2: Table S2). Notably, some of the categories are involved in the metabolic process and the regulation of skeletal muscle development.

### qPCR analysis

**Fig. 7:**
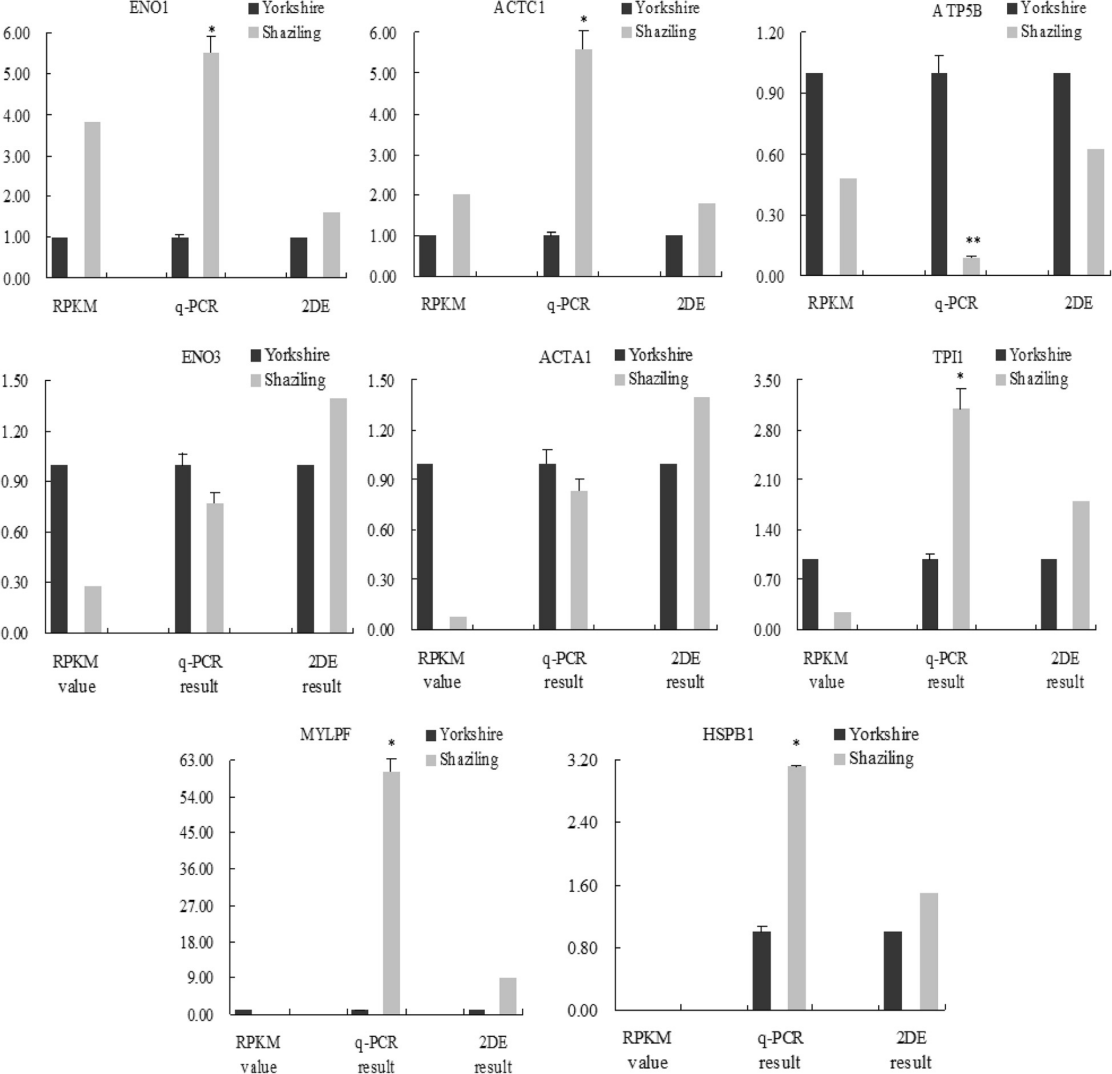
qRT-PCR validation of the differentially expressed genes analyzed by RNA-seq and 2-DE. qRT-PCR was performed for eight genes that were identified as differential expressed genes between the Yorkshire and Shaziling pig breeds. The Y axis shows the relative expression levels

To evaluate the proteomics and transcriptomics results, the expressions of eight genes were determined in the two pig breeds by qPCR on the basis of their different influence on the formation of meat flavor and skeletal muscle development. Four of the genes encoded proteins involved in glycolysis and fat deposition (*ENO1*, *ENO3*, *ATP5B*, and TPI1), three encoded proteins that correlated with synthesis of muscle fiber proteins (*MYLPF*, *ACTA1*, and *ACTC1*) and the *HSPB1* gene, whose encoded protein plays a crucial role in maintaining cellular homeostasis [54] and protects against stress [23]. The fold-changes in expression of the eight genes were compared with the transcriptomics and proteomics analysis results. As shown in Fig. 7, the qPCR results revealed that five genes were in agreement with the RNA-Seq results and six genes showed a similar trend to proteomics results. In the present research, we also observed different expression trends between the proteomics and transcriptome analyses.

### Correlation analysis of mRNA and protein expression

RNA-seq analyses identified 488 DEGs (Additional file 3: Table S3), of which 297 were upregulated in the Shaziling pig and 192 in the Yorkshire pig. Proteomics revealed identified 38 differentially abundant proteins, of which 27 protein spots were upregulated in the Shaziling pig and 11 in the Yorkshire pig. Similar to previous reports, the transcriptomics and proteomics data were divergent. In the present study, *ENO1* and *ACTC1* were overexpressed in Shaziling pigs, and *ATP5B* was overexpressed in Yorkshire pigs: only for these three genes were the proteomics results consistent with the transcriptomic results. In 2009, Timperio and colleagues performed a comparative analysis of proteomics and transcriptomics from the livers of Chianina and Holstein Friesian cattle. The results indicated that only three of 39 differentially abundant proteins were validated by microarray analyses [55]. Other research also confirmed that proteomics and transcriptomics data seldom overlap [56]. These differences were probably caused by alternative splicing, differential regulation of translation, and annotation errors of databases [57]. Another aspect concerning little overlap between transcriptomics and proteomics data is biological factors [58]. Although proteomics and transcriptomics data have almost no overlap, interaction pathway analyses could indicate shared biological significance [33]. Taking this into consideration, the differentially abundant proteins and DEGs that converged in the same metabolic pathways, especially regulation of skeletal muscle development, were meaningful. Some of the proteins and upregulated gene transcripts in Shaziling pigs were found to be involved in the same metabolic pathways, particularly the glycolytic pathway (ENO1, TPI1, and HSPB1). ENO1, a glycolytic enzyme, is positively correlated with meat tenderness [59]. TPI is also a glycolytic enzyme, and has been shown to correlate with meat tenderness in porcine muscles [60]. Notably, although the TPI1 result of proteomics and transcriptomics data did not match, pathway analyses of either DEG transcripts or proteins for the Shaziling samples were involved in a metabolic network. HSP proteins are related to protein folding and the oxidative stress response. In our research, HSPB1 was overexpressed in Shaziling pigs, and might be positively correlated with meat quality, which agreed with previous studies [61, 62]. Shaziling pigs have excellent meat quality like other Chinese indigenous pig breeds. The IMF content in Shaziling pigs is 3.5 %, in Jinhua pigs it is 3.38 %, and in Lantang pigs it is 2.46 %. By contrast, Yorkshire pigs and Landrace pigs have IMFs of 1.79 % [7, 63]. Increased IMF content can improve meat quality significantly, especially in terms of tenderness [64]. According to our results, genes related to tenderness have a higher level of expression in Shaziling pigs than in Yorkshire pigs, for example TPI1, HSPB1, and ENO1. Further analysis of the DEGs identified a number of novel genes and pathways (Additional file 1: Tables S1, Additional file 2: Table S2 and Additional file 3: Table S3), which have not been reported to affect meat quality previously. Further characterization of these novel genes might reveal the regulatory mechanism underlying meat quality.

## Conclusion

The object of this study was to investigate differences in the growth and development of skeletal muscle between Shaziling and Yorkshire pigs. The combined use of proteomic and transcriptomic analyses was effective in detecting DEGs and proteins. As a result, 38 differentially abundant proteins and 488 DEGs were identified by mass spectrometry and RNA-seq analysis, respectively. Some of the proteins and unigenes are associated with lipid metabolism or glycolytic metabolism, according to previously published results. Based on the putative results of GO term enrichment and KEGG pathway analyses, we determine that many of the differential abundant proteins and DEGs are related to lipid mobilization, energy metabolism, the cytoskeleton, and signal transduction. Our study provided valuable information that could contribute to a deeper understanding of the molecular mechanisms regulating the development and formation of skeletal muscle.

## Abbreviations

2-DE, two-dimensional fluorescence difference gel electrophoresis; DEGs, differentially expressed genes; GO, gene ontology; IMF, intramuscular fat; KEGG, Kyoto Encyclopedia of Genes and Genomes; LM, *longissimus dorsi* muscle; MS, mass spectrometry; NGS, next-generation sequencing; qPCR, quantitative real-time PCR

## Additional files

Additional file 1: Table S1. KEGG Pathway information of unigenes. (XLS 705 kb) Additional file 2: Table S2. GO enrichment of DEG. (XLS 77 kb) Additional file 3: Table S3. Summary of the differently expressed genes. (XLS 58 kb)
